# ORF3a is a key driver of maternal SARS-CoV-2 infection-associated placental dysfunction

**DOI:** 10.21203/rs.3.rs-6857689/v1

**Published:** 2025-07-03

**Authors:** Indira Mysorekar, Deepak Kumar, Eliza McColl, Rowan Karvas, Brittany Jones, Long Tran, Emily Diveley, Sukanta Jash, Surendra Sharma, Jeannie Kelly, Thorold Theunissen

**Affiliations:** Baylor College of Medicine; Baylor College of Medicine; Baylor College of Medicine; University of Colorado, Anschutz Medical Campus; Baylor College of Medicine; Baylor College of Medicine; Washington University School of Medicine; Brown University; UTMB Galveston; Washington University School of Medicine; Washington University School of Medicine

**Keywords:** tight junctions, placenta, COVID-19, PDZ domain, intrinsically disordered protein, CD63

## Abstract

SARS-CoV-2 infection during pregnancy is associated with an increased risk of pre-eclampsia (PE), a hypertensive disorder, but the molecular mechanisms remain poorly understood. Here, we identify ORF3a, a SARS-CoV-2 accessory protein, as a key factor in placental dysfunction, driving autophagy dysregulation, trophoblast maturation impairment, protein aggregation and placental barrier disruption–processes linked to PE. We detect ORF3a in placentas from women infected with SARS-CoV-2 along with increased protein aggregation and disrupted tight junctions in ORF3a + regions. In placental cell lines, ORF3a impairs syncytiotrophoblast maturation and induces protein aggregation. Mechanistically, ORF3a binds to ZO-1 via its PDZ-binding motif (SVPL), and deletion of this domain from ORF3a abrogates its effect on trophoblast barrier integrity. In human trophoblast cells engineered with an LC3-GFP-mCherry reporter, ORF3a induces autophagosome accumulation, and shifts autophagy toward a secretory pathway with elevated levels of CD63 + extracellular vesicles and disrupted ZO-1 localization, all of which are recapitulated by live infection with the SARS-CoV-2 Delta variant. These ORF3a-dependent changes are fully recapitulated in 3D stem-cell-derived trophoblast organoids (SC-TOs). Together, our findings define a molecular mechanism by which SARS-CoV-2 infection compromises placental syncytial integrity. Targeting ORF3a may provide a therapeutic strategy to mitigate PE-like placental dysfunction in SARS-CoV-2–infected pregnancies.

## Introduction

The pandemic triggered by severe acute respiratory coronavirus 2 (SARS-CoV-2) has resulted in over 7 million fatalities as of 2024^1^. Although global vaccination efforts have mitigated the severity of COVID-19^2–4^, maternal infection during pregnancy has been consistently associated with a spectrum of adverse outcomes such as preeclampsia (PE), and long-term neurodevelopmental risks in offspring^5–12^. During the peak of the pathogenic Delta variant outbreak, there was a significant increase in adverse pregnancy outcomes^11,13^. Despite rare vertical transmission^14,15^, placental pathologies and adverse pregnancy outcomes persist even after infections during pregnancy resolve^16,17^, indicating that the effects of SARS-CoV-2 infection can be long-lasting. Recently, long COVID has been identified as a novel multi-organ condition^18,19^, and can potentially impact women post-partum^20–22^. The emergence of the new immune-evasive variant (NB.1.8.1) has reactivated concerns about the vulnerability of pregnant women^23^. Given generally low acceptance of vaccines against SARS-CoV-2 in pregnant women and new policy changes recommending against the necessity of COVID-19 immunization in this vulnerable population^24^, there remains an unmet need to understand how SARS-CoV-2 affects pregnancy and causes both short- and long-term sequelae to develop alternate therapeutic strategies.

The placenta serves as a physical and immunological barrier between the mother and the fetus^25,26^. Placental sufficiency requires maturation of cytotrophoblasts (CTBs) into human chorionic gonadotropin (hCG)-β-secreting syncytiotrophoblasts (STBs), which play an essential role in immune defense and nutrient and gas exchange between mother and fetus, as well as into extra-villous trophoblasts (EVT) whose migration is crucial for proper remodeling of maternal blood vessels. However, these placental cells express the SARS-CoV-2 entry receptor, ACE2, and we and others have observed SARS-CoV-2 proteins and viral RNA in different compartments of the placenta during the course of gestation^27,28^. Thus, despite low rates of vertical transmission, this demonstrates that SARS-CoV-2 can successfully invade the placenta and could have lasting effects on placental biology and function. However, the exact mechanisms through which SARS-CoV-2 impacts placental function remain unclear.

Autophagy, a crucial physiological process, plays a vital role in placental development by maintaining cellular homeostasis, and its dysregulation is associated with pregnancy complications such as preterm birth, miscarriage, and growth restriction^29,30^. It also regulates epithelial barrier integrity by controlling tight junction protein (TJP) turnover^31,32^. While autophagy disruption has been observed in the lungs and immune cells of COVID-19 patients^33–35^, its impact on the placenta remains unclear. When autophagosome-lysosome fusion is blocked—such as by SARS-CoV-2—cells may shift to secretory autophagy, releasing undigested cargo via extracellular vesicles^36–42^.

The accessory protein ORF3a of SARS-CoV-2 has become a significant pathogenic element in the pathophysiology of SARS-CoV-2 and, similar to Spike protein, exhibits considerable mutability^41,43^: approximately 175 mutations in the ORF3a protein have been identified across different SARS-CoV-2 variants^44^. ORF3a of SARS-CoV-2 has been shown to inhibit autophagosome-lysosome fusion and altered lysosomal egress^45^. Further, ORF3a harbors an intrinsically disordered C-terminus with a PDZ-binding motif (PBM), a short linear protein sequence (SVPL) with high binding affinity to TJPs such as ZO-1, which contain PDZ-binding domain motifs^46^.

Here, we show that SARS-CoV-2 ORF3a is present in placental tissue from term deliveries of infected pregnant women. ORF3a+ placental regions exhibit protein aggregation and disrupted TJP. Infected placentas exhibit evidence of protein aggregation and disrupted TJPs in ORF3a+ regions. Infection of placental cells with live SARS-CoV-2 (Delta variant) impairs autophagic flux, a mechanism that is recapitulated by ORF3a expression alone in placental cell lines and three-dimensional (3D) stem-cell-derived trophoblast organoid (SC-TO) models. This disruption impairs STB maturation and EVT trophoblast invasion and leads to aberrant protein aggregation. We further demonstrate that ORF3a activates secretory autophagy and increases production of CD63+ extracellular vesicles and supernatants from ORF3a+ cells alter autophagy in recipient cells. Further, SARS-CoV-2 Delta compromises CTB barrier integrity through interaction of ORF3a with ZO-1 via its PBM. Deleting this motif restores ZO-1 localization and alters ORF3a association with CD63, indicating the importance of the PBM motif in loading on CD63+ vesicles. Together, our findings highlight a molecular and cellular mechanism through which SARS-CoV-2 ORF3a disrupts placental syncytial integrity.

## Results

### ORF3a impairs autophagic flux in placental trophoblasts

**Figure 1:**
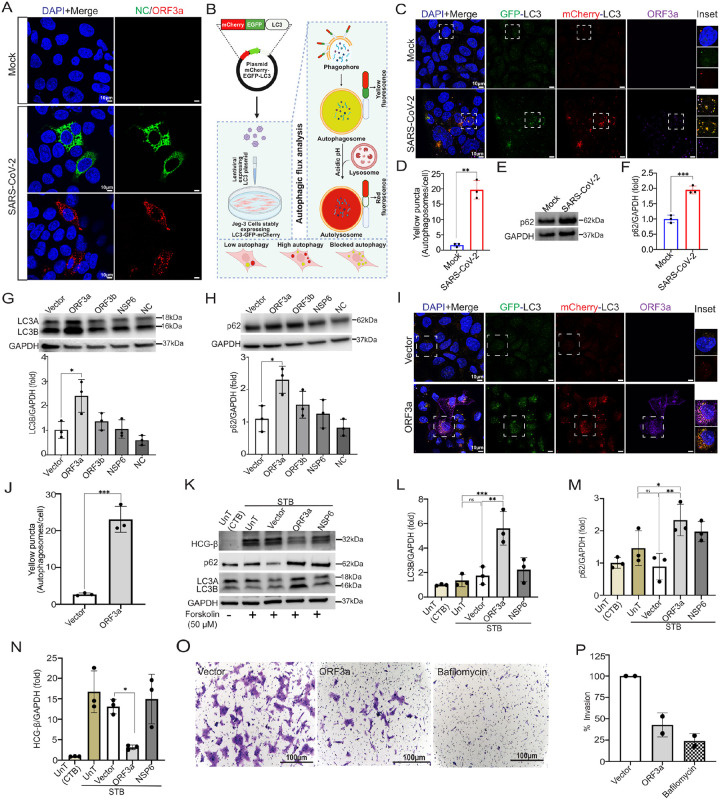
ORF3a blocks autophagy flux in CTBs, STBs and reduces invasion in EVTs. **(A)** JEG-3 cells were infected by SARS-CoV-2 Delta at an MOI of 1 and immunostained with Nucelocapsid (green) and ORF3a (red). Scale bar, 10 μm. **(B)** Schematic diagram of JEG-3 cell line stably expressing LC3-EGFP-mCherry c and suggesting labelled autophagosome yellow when not fused with lysosomes and becomes red when fused with lysosomes. **(C)** Representative confocal images of JEG-3-LC3-EGFP-mCherry cells infected with SARS-CoV-2 Delta variant (MOI 1), showing LC3 tagged with EGFP (green) and mCherry (red), and stained for ORF3a (purple). Merged images with DAPI (blue) highlight autophagosomes as yellow puncta (co-localization of EGFP and mCherry). Regions where yellow autophagosomes overlap with ORF3a appear white. Insets magnify areas showing this co-localization. Scale bar, 10 μm. **(D)** Quantification of autophagosomes (yellow puncta) per cell from three independent experiments (n = 75 cells total). Data are presented as mean ± SD. p < 0.01, unpaired two-tailed Student’s t test. **(E)** JEG-3 cells infected with Delta for 48 h, followed by Western blot for p62 protein expression. **(F)** Densitometric quantification of p62 protein expression normalized to GAPDH as loading control using Image Lab software (Data of three independent experiments were represented as mean ± SD, ***p ≤ 0.001; unpaired two-tailed Student’s t test). **(G)** JEG-3 cells transfected with SARS-CoV-2-encoded plasmids for 24 h, followed by Western blot for LC3B protein expression. Densitometric quantification of LC3B protein expression normalized to GAPDH as loading control using Image Lab software (Data of three independent experiments were represented as mean ± SD, *p ≤ 0.05; ANOVA comparison test). **(H)** p62 protein expression detection by Western blot from JEG-3 transfected cells, followed by densitometric quantification of data acquired from three independent experiments (mean ± SD, *p ≤ 0.05; ANOVA comparison test). **(I)** Representative confocal images of JEG-3-LC3-EGFP-mCherry cells transfected with ORF3a and only vector plasmids, showing LC3 tagged with EGFP (green) and mCherry (red), and immunostained for ORF3a (purple). Merged images with DAPI (blue) display autophagosomes as yellow puncta (co-localization of EGFP and mCherry). Regions where yellow autophagosomes co-localize with ORF3a appear white. Insets highlight examples of yellow autophagosomes and their overlap with ORF3a. **(J)** Quantification of autophagosomes (yellow puncta) per cell from three independent experiments (n = 75 cells total). Data are presented as mean ± SD. p < 0.001, unpaired two-tailed Student’s t test. **(K)** A representative Western blot of forskolin (50μM) treated JEG-3 cells as STB models which are also transfected with SARS-CoV-2 plasmids for 24 h and analyzed for expression of LC3B, p62 and HCG- β. Expression of LC3B **(L)** and p62 **(M)** increases significantly in ORF3a transfected STBs whereas HCG-β **(N)** shows reduced expression (Data of three independent experiments were represented as mean ± SD, *p ≤ 0.05, **p ≤ 0.01and ***p ≤ 0.001, Tukey’s multiple comparisons test). **(O)** Brightfield microscopy after crystal violet staining of HTR-8 cells transfected with SARS-CoV-2 plasmids, also treated with bafilomycin for Matrigel invasion assay. **(P)** The quantification invasion was represented as percentage calculated after counting number of cells that invaded the Matrigel membrane from two independent experiments, analyzing a total of 10 regions of interest (ROIs).

**Figure 2:**
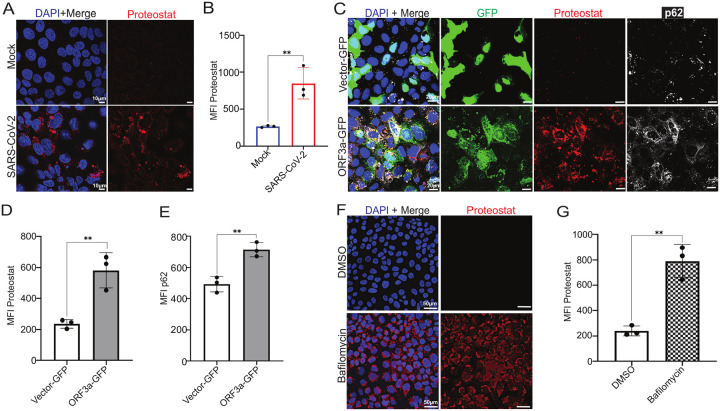
ORF3a induces protein aggregation. **(A)** Representative confocal images of JEG-3 cells mock-infected or infected with SARS-CoV-2 Delta variant, stained with ProteoStat (red) to detect protein aggregates. Nuclei were counterstained with DAPI (blue). **(B)** Quantification of mean ProteoStat intensity was performed across three independent experiments, after averaging mean fluorescence intensity (MFI) from a minimum of six images per condition and per biological replicate and shown as mean ± SD, **p < 0.01, using unpaired two-tailed Student’s t test. **(C)** Representative confocal images of JEG-3 CTBs transfected with ORF3a-GFP (green) exhibit enhanced proteostat staining (red) and increased p62 staining (white) relative to Vector-GFP transfected CTBs. observed colocalization with proteostat and ORF3a-GFP. Scale bar, 20 μm. **(D)** and **(E)** Data quantification for proteostat and p62 respectively, from three independent experiments for panel after averaging mean fluorescence intensity (MFI) from a minimum of six images per condition and per biological replicate and shown as mean ± SD, **p < 0.01, using unpaired two-tailed Student’s t test. **(F)** Confocal imaging of JEG-3 cells treated with bafilomycin and DMSO (vehicle control) and stained with proteostat dye (red). Scale bar, 50 μm. **(G)** Mean fluorescence intensity (MFI) quantification shows increased proteostat staining in bafilomycin treated JEG-3 cells, data of three independent experiments with averaging MFI from minimum of five images each experiment, mean ± SD, **p < 0.01, unpaired two-tailed Student’s *t* test.

To examine the presence and potential impact of SARS-CoV-2 in the placenta, we performed immunostaining on term placental villous tissue from gestational age-matched uninfected and SARS-CoV-2-infected women, including individuals who tested positive during the first trimester. ORF3a was detected in placentas from infected pregnancies, notably within syncytiotrophoblasts (STBs) (**Supp**. Figure 1A). The persistence of this accessory viral protein at term suggests prolonged viral colonization and possible replication in placental tissue, even in the absence of active maternal infection at delivery—consistent with previous reports^27,47,48^. To investigate how SARS-CoV-2 infection may alter trophoblast function, we infected JEG-3 cells, a model of cytotrophoblasts (CTBs)^49^, with the Delta variant of the virus. Immunostaining 48 hours post-infection indicated the presence of both viral nucleocapsid and ORF3a proteins, consistent with active viral protein expression (Fig. 1A). Viral titers of infected JEG-3 cells showed low (**Supp**. Figure 2) but efficient viral replication, consistent with recent studies^50^.

We next assessed whether SARS-CoV-2 infection alters autophagy in placental trophoblasts, a process critical for trophoblast function and placental development. To examine this, we generated a reporter JEG-3 cell line stably expressing LC3, an autophagy marker protein, dual-tagged with fluorescent proteins, mCherry and EGFP (Fig. 1B). Yellow puncta (merged EGFP and mCherry) indicate autophagosome formation, while red-only puncta reflect successful fusion with lysosomes, where the acidic environment quenches EGFP fluorescence. In this system, under basal conditions, we would expect to observe only a few yellow or red vesicles, whereas an increase in autophagy would be marked by a predominance of red puncta, and a block in autophagic flux would lead to the accumulation of yellow puncta, reflecting impaired autophagosome–lysosome fusion (Fig. 1B). We validated these observations in the autophagy reporter cell line using bafilomycin, an autophagy flux inhibitor, and metformin, an autophagy inducer (**Suppl. Figure 3**). Bafilomycin treatment led to the accumulation of yellow vesicles, indicating a buildup of unprocessed autophagosomes. In contrast, metformin treatment resulted in fewer yellow vesicles and an increase in red vesicles, consistent with enhanced autolysosome formation and ongoing autophagic flux. Infection of the LC3 reporter JEG-3 cell line with the SARS-CoV-2 Delta variant for 48 hours resulted in a significant increase in yellow puncta in infected cells compared to mock-infected controls, suggesting an accumulation of autophagosomes (Fig. 1C–D). Notably, cells with elevated yellow puncta also exhibited strong ORF3a staining, with clear colocalization between ORF3a and the autophagosomal yellow structures (Fig. 1C **inset**). To further probe whether this accumulation reflected impaired autophagic flux, we assessed p62 levels by western blot (Fig. 1E–F). We found that p62 expression was significantly elevated in infected trophoblasts, consistent with a SARS-CoV-2-induced block in autophagosome degradation and disrupted autophagic flux.

To elucidate whether ORF3a specifically modulates autophagy in placental cells, JEG-3 cells were transfected with plasmids encoding SARS-CoV-2 proteins representative of different classes: ORF3a, ORF3b (accessory), Nucleocapsid (N) (structural), NSP6 (non-structural), and an empty vector backbone as control. Among these, only ORF3a induced a significant increase in the autophagy markers, LC3B (Fig. 1G) and p62 (Fig. 1H), consistent with impaired autophagic flux. Although NSP6 has previously been reported to reduce autophagosome size^51^, our data show no significant effect of NSP6 on overall autophagic flux. Thus, NSP6 served as a biologically relevant control, supporting the conclusion that disruption of autophagic flux is specific to ORF3a, rather than a general property of SARS-CoV-2 proteins that interact with the autophagy pathway. Consistent with findings from live virus infection, transfection of the JEG-3-LC3-EGFP-mCherry reporter cell line with ORF3a led to a significant accumulation of yellow puncta (Fig. 1I–J). ORF3a colocalized with these yellow autophagosomal structures, suggesting a direct role in blocking autophagosome–lysosome fusion. To assess effects in differentiated STBs, we used forskolin-treated syncytialized JEG-3 cells that exhibited increased hCG-β expression (Fig. 1K), confirming syncytialization. ORF3a transfection of STBs also showed elevated LC3B and p62 (Fig. 1L–M), indicating that ORF3a, but not NSP6, disrupts autophagic flux in both CTBs and STBs. Supporting these findings, increased LC3 and p62 staining was observed in placentas from SARS-CoV-2–infected pregnancies (**Suppl. Fig. S1 B-C**). Because autophagy plays a key role in CTB-to-STB differentiation^52^, we next examined the impact of ORF3a on hCG-β expression in STBs. STBs transfected with ORF3a exhibited significantly decreased hCG-β expression (Fig. 1N), suggesting that ORF3a impairs syncytial differentiation.

We next assessed the effects of ORF3a on EVTs, which also differentiate from CTBs and perform the essential function of invading maternal decidua to remodel spiral arteries, an autophagy-dependent process^30,53^. We used HTR-8/SVneo cells, a widely used *in vitro* model representing EVTs^54^, to test whether ORF3a impairs invasion. ORF3a-transfected HTR-8 cells displayed significantly reduced invasion through Matrigel-coated transwell inserts compared to vector controls, suggesting a disruption of cell invasion mechanisms (Fig. 1O–P). Notably, EVTs treated with bafilomycin A1, an inhibitor of autophagic flux, exhibited similar impairments in invasion, supporting the conclusion that ORF3a impairs trophoblast invasion via disruption of autophagy. Together, these results demonstrate that SARS-CoV-2 ORF3a blocks autophagic flux across multiple trophoblast cell types leading to impaired differentiation and invasive function.

### ORF3a induces protein aggregation

Canonical autophagy serves a degradative function, clearing misfolded aggregated protein complexes to maintain proteostasis^55^. Disruption of this process in the placenta has been linked to protein aggregate accumulation and adverse pregnancy outcomes, including pre-eclampsia^29,56^. To determine whether SARS-CoV-2 infection leads to protein aggregation, we performed confocal imaging of JEG-3 cells infected with SARS-CoV-2 Delta variant. Infected cells displayed accumulation of Proteostat dye-positive structures, indicative of aggregated proteins^57^ (Fig. 2A–B). To ascertain whether ORF3a plays a role in the observed aggregation, we transfected JEG-3 cells with ORF3a or vector controls. ORF3a-expressing cells exhibited significantly greater accumulation of Proteostat-positive aggregates compared to controls (Fig. 2C–D). Furthermore, Proteostat-positive aggregates colocalized with p62 (Fig. 2C), which was markedly increased in ORF3a-transfected JEG-3 cells (Fig. 2E). Notably, pharmacological inhibition of autophagic flux with bafilomycin A1 similarly increased Proteostat staining (Fig. 2F–G), supporting the idea that defective autophagic degradation leads to protein aggregate accumulation. Consistent with these findings, increased Proteostat staining was also observed in placentas from SARS-CoV-2-infected pregnancies (**Sup Fig. 1D**). Together, these findings suggest that SARS-CoV-2 induces protein aggregation through ORF3a-mediated blockage of autophagic flux, leading to impaired degradation of p62-marked proteins and their subsequent accumulation.

### ORF3a-mediated disruption of canonical autophagy activates secretory autophagy and enhances extracellular vesicle (EV) release

**Figure 3:**
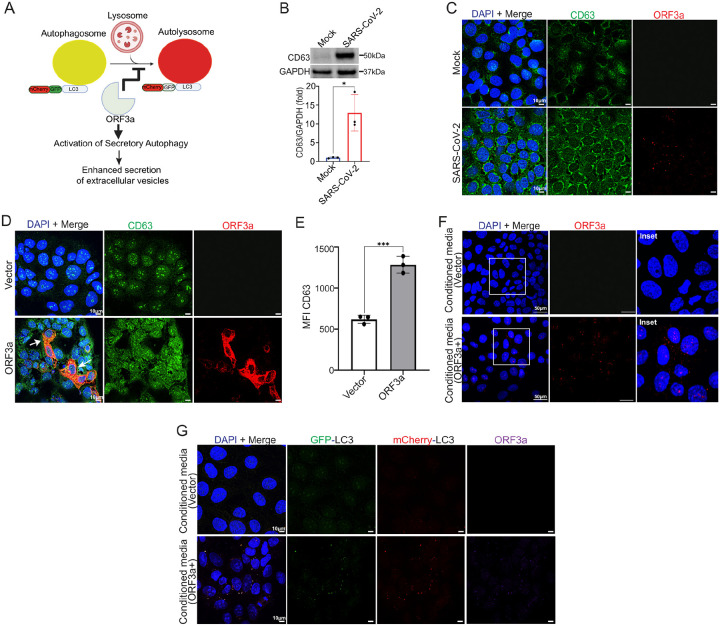
ORF3a induces secretory autophagy and is secreted with EVs. **(A)** Schematic diagram showing ORF3a impact of blocking autophagosome fusion with lysosome leads to activation of secretory autophagy and enhanced extracellular vesicle secretion. **(B)** A representative Western blot of JEG-3 cells infected with Delta (MOI 1) for 48 h showing increased CD63 expression as fold change from mock infection (n=3, mean ± SD, **p < 0.01, unpaired two-tailed Student’s t test). **(C)** Representative confocal images of JEG-3 cells infected with Delta showing CD63 (green) and ORF3a (red), nuclei stained with DAPI. Scale bar, 10 μm. **(D)** Confocal representative IF images from three independent experiments show enhanced formation of CD63+ (green) vesicles in ORF3a-WT transfected (red) JEG-3 cells as compared to vector control and yellow color shows colocalization in Merge panel (Scale bar = 50 μm). **(E)** Comparison of CD63+ vesicles MFI between vector and ORF3a-WT transfected cells with significant differences (mean ± SD,***p<0.001) determined by unpaired two-tailed Student’s t test, based on 5 images per condition per group from three independent experiments. **(F)** Confocal IF images from three independent experiments show JEG-3 cells treated with conditioned media from Vector and ORF3a-WT (red) transfected cells, with nuclei stained using DAPI (Scale bar = 50 μm). **(G)** Conditioned media form JEG-3-LC3-EGFP-mCherry cells transfected with ORF3a and vector incubated on healthy JEG-3 cells showing Merge panel with yellow (GFP-LC3 + mCherry-LC3) autophagosome and separate panels with GFP-LC3 (green), mCherry-LC3 (red) and ORF3a (purple) (Scale bar, 10 μm).

When autophagosome–lysosome fusion is blocked or lysosomal function is impaired, autophagy can shift from a degradative to a secretory mode, in which autophagosomes or dysfunctional lysosomes traffic to the plasma membrane and release their cargo into the extracellular space^39,58^ (Fig. 3A). Given our observations that infection with SARS-CoV-2 Delta variant impairs canonical autophagic flux through ORF3a, we investigated whether this triggers secretory autophagy in trophoblasts. CD63 is a well-established marker of EVs generated through secretory autophagy^59,60^; thus, we examined CD63 expression and vesicle production following infection. Indeed, SARS-CoV-2 Delta-infected JEG-3 cells had significantly higher CD63 expression (Fig. 3B), and immunofluorescence revealed a marked increase in CD63 + vesicles in infected cells, consistent with activation of the secretory autophagy pathway (Fig. 3C). Similarly, JEG-3 cells transfected with ORF3a exhibited increased CD63 expression compared to vector-transfected controls (Fig. 3D–E). Immunofluorescence revealed colocalization of ORF3a with CD63^+^ structures (Fig. 3D), suggesting that ORF3a may be incorporated into and secreted via CD63^+^ extracellular vesicles during secretory autophagy. To test this, conditioned media from ORF3a-transfected JEG-3 cells were harvested, cleared of any cellular debris, and added to naïve, non-transfected JEG-3 cells. Confocal microscopy revealed robust ORF3a staining in recipient cells (Fig. 3F), indicating that ORF3a was secreted into the media and taken up by neighboring cells.

To determine whether unfused autophagosomes were also secreted, we transfected the JEG-3-LC3-EGFP-mCherry reporter cell line with ORF3a and collected the conditioned media 24 hours after transfection (Fig. 3G). When this media was applied to naïve, non-fluorescent JEG-3 cells, recipient cells developed yellow LC3 puncta that colocalized with ORF3a, indicating uptake of autophagosome components along with ORF3a. These results suggest that secretory autophagy is activated in the context of ORF3a-induced autophagy blockade, resulting in the release of ORF3a and autophagic cargo in CD63 + vesicles, which can be taken up by neighboring cells, even in the absence of active viral replication.

### ORF3a interacts with ZO-1 via its PDZ-binding motif and disrupts tight junction organization in trophoblasts

**Figure 4:**
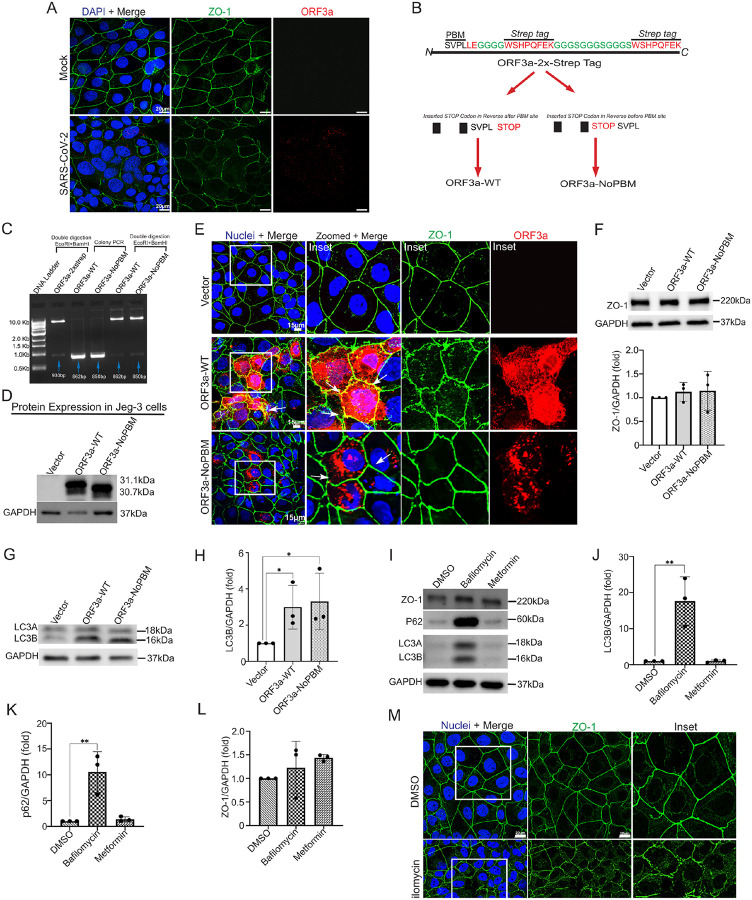
ORF3a associates with ZO-1 via PBM motif and alters its localization. **(A)** JEG-3 cells infected with Delta (MOI 1) showing ZO-1 (green) and ORF3a (red), nuclei blue with DAPI. Scale bar, 20μm. **(B)** Schematic illustration of the constructed plasmids: ORF3a-WT (wild type or untagged) and ORF3a-NoPBM (with PBM motif deletion). **(C)** Clone confirmation by DNA gel electrophoresis demonstrates that colony PCR from positive colonies successfully integrated ORF3aWT (862 bp) and ORF3a-NoPBM (850 bp) genes at their respective sizes into the parent ORF3a-2X-Strep plasmid. This was further verified by double digestion, confirming the correct integration of genes with the pLVX backbone plasmid. **(D)** Western blot analysis of ORF3a-WT and ORF3a-NoPBM expression in transfected JEG-3 cells. **(E)** Immunostaining of JEG-3 cells transfected with vector control, ORF3a-WT and ORF3a-NoPBM shows co-localization of ORF3a-WT (in red) and ZO-1 (in green) as compared to ORF3a-NoPBM and Vector. Insets provide magnified views of the co-localization regions (Scale bar = 15μm). **(F)** Western blot of Jeg-3 cells transfected with ORF3a-WT and ORF3a-NoPBM show unchanged expression of ZO-1 as represented by data of three independent experiments with mean ± SD, p>0.05 as non-significant. **(G)** and**(H)** LC3B expressions analyzed through western blot show increased trend in both ORF3a-WT and ORF3a-NoPBM. (Data presented as mean ± SD, *p ≤ 0.05) **(I)** JEG-3 cells treated with bafilomycin, metformin and respective vehicle control DMSO, analysed for expression levels of LC3B, p62 and ZO-1. (mean ± SD **p ≤ 0.01)**(J)** Quantification of LC3B expression, **(K)** p62 expression and **(L)**ZO-1 expression in bafilomycin and metformin treatment. **(M)** Immunostaining and confocal imaging for JEG-3 cells treated with DMSO, bafilomycin and metformin, representative images from three independent experiments show expression of ZO-1 (green) and nuclei stained with DAPI (Scale bar = 20μm).

Canonical autophagy plays a role in preserving the structural integrity of epithelial cell-cell junctions by controlling the trafficking and recycling of TJPs, such as ZO-1^39^. Given our findings that ORF3a induces a transition from conventional autophagy to secretory autophagy, we next examined the influence of ORF3a on trophoblast barrier integrity. JEG-3 cells infected with SARS-CoV-2 Delta variant exhibited altered ZO-1 staining in regions positive for ORF3a, suggesting disruption of tight junction architecture (Fig. 4A).

ORF3a harbors a short linear motif (SLiM) designated PBM (PDZ binding motif) (amino acid sequence *SVPL*) at its cytosolic C-terminus (Fig. 4B). PBMs are known to interact with host PDZ domain-containing proteins that play a role in cell-cell junctions and polarity^61^. Thus, we hypothesized that ORF3a may directly bind to the PDZ domain of ZO-1 via its C-terminal PBM. However, the commercially available pLVX-EF1alpha-ORF3a-2xStrep plasmid has a 2xStrep tag on the C-terminus of ORF3a, which would likely obstruct PBM-mediated interactions (Fig. 4B). To enable PBM accessibility, we used restriction enzyme-based cloning to eliminate the 2x-Strep tag by inserting a stop codon upstream of the tag sequence and introducing EcoRI and BamHI restriction sites, generating an untagged wild-type construct (hereafter, **ORF3a-WT**). We also generated a PBM-deletion mutant (hereafter, **ORF3a-NoPBM**) by inserting a stop codon immediately upstream of the PBM, effectively truncating the PBM, and added a BamHI site after the stop codon. Primers were designed to incorporate an EcoRI site in the forward direction. The resulting amplicons were digested with EcoRI and BamHI, ligated into the pLVX-EF1α vector, and validated by colony PCR, restriction digest, and Sanger sequencing (Fig. 4C).

Newly generated plasmids ORF3a-WT and ORF3a-NoPBM successfully expressed either form of ORF3a when transfected into JEG-3 cells (Fig. 4D). In cells expressing ORF3a-WT, strong colocalization with ZO-1 was observed (Fig. 4E; **inset**), consistent with observations in SARS-CoV-2-infected human placenta samples (**Supp**. Figure 1E). In contrast, ORF3a-NoPBM exhibited minimal co-localization with ZO-1 (Fig. 4E; **inset**). Furthermore, while the overall protein expression of ZO-1 was unchanged by either form of ORF3a (Fig. 4F), ORF3a-WT caused mis-localization of ZO-1 and breakage of tight junctions while the PBM mutant did not (Fig. 4E; **inset**). These findings suggest that ORF3a disrupts tight junction organization through direct interaction with ZO-1, and that this interaction requires the PBM domain. Additionally, while ORF3a-WT was distributed throughout the cell, the ORF3a-NoPBM mutant was less widely distributed and only present in perinuclear regions (Fig. 4E; **Inset**), suggesting that the PBM domain of ORF3a may also play a role in intracellular transport of ORF3a.

We next sought to determine whether ORF3a-induced disruption of ZO-1 localization is solely due is solely due to direct interaction via its PBM, or whether it is also influenced by ORF3a-mediated blockade of autophagic flux. We observed that both ORF3a-WT and ORF3a-NoPBM significantly increased LC3B expression (Fig. 4G and 4H), indicating that both constructs impair autophagic flux. These findings suggest that ZO-1 mislocalization may result from a combination of direct binding by ORF3a and indirect effects through autophagy disruption. Thus, to investigate the potential indirect regulation of ZO-1 expression and localization by autophagy, JEG-3 cells were treated with either bafilomycin (autophagy inhibitor) or metformin (autophagy activator)^37^. Bafilomycin treatment did not alter overall ZO-1 protein levels (Fig. 4I–L), but increased LC3B and p62 expression, confirming impaired autophagic flux (Fig. 4J–K). Under these conditions, ZO-1 was mislocalized away from the membrane (Fig. 4M), phenocopying the effect of ORF3a-WT. In contrast, metformin treatment restored autophagic flux, normalized LC3B and p62 levels, and preserved ZO-1 localization at cell–cell junctions (Fig. 4M). Together, these results indicate that ORF3a-mediated disruption of ZO-1 localization involves both direct interaction via its PBM and autophagy-dependent mechanisms.

### ORF3a impairs trophoblast maturation and promotes EV secretion in 3D stem cell–derived trophoblast organoids (SC-TOs)

**Figure 5:**
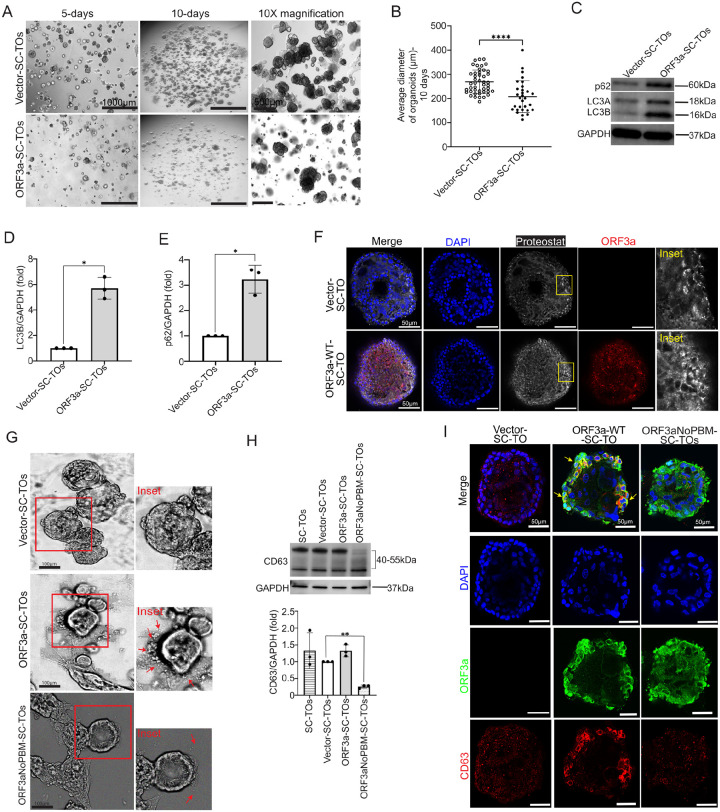
ORF3a reduces maturation and induces secretion of EVs in 3D SC-TOs. **(A)** Bright field images captured at 5x and 10x magnification of SC-TOs developed from CT30-Vector and CT30-ORF3aWT, demonstrating growth at 5 and 10 days (Scale Bar, 1000 and 500 μm). **(B)** Comparison of average organoid diameter between CT-30-Vector and CT-30-ORF3a-WT, with significant difference (mean ± SD, ****p<0.0001) determined by Mann-Whitney test, based on nearly 30 ROIs per group. **(C)** A representative Western blot of proteins isolated from vector-SC-TOs and ORF3a-WT-SC-TOs probed for LC3B and p62 expression. **(D)** and **(E)** represents the quantification of blot showing increased expression of LC3B and p62 in ORF3a-WT-SC-TOs (data represented from three independent experiments as mean ± SD, *p < 0.05) **(F)** Representative confocal images of vector-SC-TO and ORF3a-WT-SC-TO samples stained with ProteoStat (white), DAPI (blue), and immunostained for ORF3a (red). Merged images show differential accumulation of protein aggregates between the two groups. Insets highlight regions where increased ProteoStat signal (white) is observed, with yellow coloration indicating areas of overlap. Scale bar, 50 μm. **(G)** Brightfield images of SC-TOs at 20x magnification illustrating the formation of extracellular vesicles (inset: red arrows) by CT-30-ORF3a-WT organoids (Scale Bar=100 μm). **(G)** Representative Western blot showing expression of CD63 where quantification **(H)** revealed reduced expression of CD63 in ORF3a-NoPBM-SC-TOs. **(I)** Representative confocal images of Vector-SC-TO, ORF3a-WT-SC-TO, and ORF3a-NoPBM-SC-TO samples immunostained for CD63 (red) and ORF3a (green). Merged images show differential localization and co-distribution of CD63-positive vesicles (yellow arrow) with ORF3a. Scale Bar, 50 μm.

To assess the effects of ORF3a on autophagy and trophoblast differentiation in a more physiologically relevant model, we employed 3D human stem-cell–derived trophoblast organoids (SC-TOs)^62–64^. To evaluate the effect of ORF3a on 3D development of SC-TOs, we generated CT30 human trophoblast stem cell (hTSC) lines stably expressing ORF3a-WT, ORF3a-NoPBM, or an empty vector via lentiviral transduction. These engineered hTSCs were then used to establish SC-TOs using our previously published protocols^62^. Organoid development was monitored by bright-field imaging on days 5 and 10 to assess morphological progression. Our findings revealed that SC-TOs derived from ORF3a-expressing hTSCs exhibited impaired growth and maturation compared to vector controls (Fig. 5A). Notably, the average diameter of ORF3a-expressing SC-TOs was significantly smaller than that of the vector control SC-TOs at both observed time points (Fig. 5A–B). Furthermore, autophagic flux analysis showed increased expression of LC3b and p62 in ORF3a-expressing SC-TOs as compared to vector controls (Fig. 5C–E), supporting disruption of autophagic degradation, consistent with our findings in placental cell lines. Immunostaining further showed increased Proteostat signal in ORF3a-expressing SC-TOs, indicating accumulation of aggregated proteins due to blocked autophagy (Fig. 5F). Moreover, we found that ORF3a-SC-TOs are encircled by numerous extracellular vesicles (EVs) (Fig. 5G), suggesting activation of secretory autophagy in this 3D system. Although ORF3a-NoPBM–SC-TOs also secreted EVs, this occurred at a lower frequency, implying that the PBM domain enhances secretory output. Interestingly, unlike in 2D cell cultures, CD63 protein levels in ORF3a-SC-TO lysates were similar to vector controls. However, expression of ORF3a-NoPBM resulted in a significant reduction of CD63 levels, specifically impacting higher molecular weight isoforms (Fig. 5H). CD63, a heavily glycosylated tetraspanin, exists in multiple isoforms that migrate differently (typically between 40–55 kDa) on SDS-PAGE depending on their glycosylation status^65^. The apparent loss of these glycosylated isoforms in ORF3a-NoPBM–expressing SC-TOs suggests that the PBM domain may influence CD63 glycosylation or trafficking. Finally, immunofluorescence revealed colocalization of wild-type ORF3a with CD63 + vesicles in SC-TOs, whereas ORF3a-NoPBM showed no such association (Fig. 5I). Together, these findings suggest that ORF3a impairs SC-TO growth, disrupts autophagic flux, promotes protein aggregation, and enhances versicle secretion. These effects are partially dependent on the PBM domain, which also appears to regulate CD63 association and glycosylation, highlighting a key role for ORF3a in modulating both intracellular trafficking and intercellular communication in 3D trophoblast organoids.

## Discussion

There remains a substantial gap in understanding the molecular mechanisms by which SARS-CoV-2 disrupts placental function and contributes to adverse pregnancy outcomes. Our findings reveal a previously unrecognized pathway by which the SARS-CoV-2 accessory protein ORF3a impairs key placental functions, including syncytialization, trophoblast invasion, and barrier integrity—through blockade of canonical autophagy and induction of secretory autophagy, resulting in elevated extracellular vesicle signaling. While prior studies have shown that ORF3a can inhibit autophagic flux in various cell types^66–68^, our work extends this knowledge by demonstrating functional consequences of this disruption in the context of human placental biology. Specifically, we show that ORF3a modulates processes closely linked to placental dysfunction and preeclampsia, characterized by impaired syncytiotrophoblast differentiation, shallow trophoblast invasion and disrupted placental barrier integrity^10,69^. Many of these hallmarks were recapitulated by ORF3a expression in both placental cell lines and physiologically relevant 3D stem-cell–derived trophoblast organoids. Importantly, we detected ORF3a protein in human placentas from SARS-CoV-2 infected pregnancies, together with protein aggregation and tight junction disruption in ORF3a + regions. These *in vivo* observations align with our mechanistic findings and suggest that ORF3a may contribute to PE-like placental dysfunction in SARS-CoV-2 infected pregnancies. Notably, the ability of ORF3a to both block autophagy and directly interact with ZO-1 through its PBM motif provides a dual mechanism for disrupting trophoblast barrier function, a critical process for fetal protection and immune modulation at the maternal-fetal interface.

Aberrant autophagy disruption and consequent protein aggregation have been implicated in the pathogenesis of severe PE^29,56,70,71^. Consistent with this, we show that both Delta variant infection and ORF3a expression alone induce Proteostat-positive protein aggregates, a pathological feature correlated with PE severity^29,56^. Importantly, similar aggregates were detected in SARS-CoV-2–exposed term placentas, suggesting that ORF3a-triggered autophagy defects and protein aggregation may persist even after viral clearance, with potentially lasting impacts on placental health. This provides a plausible mechanistic link to the increased incidence of PE observed following SARS-CoV-2 infection during pregnancy^72,73^. Notably, despite limited productive Delta infection in JEG-3 trophoblasts—consistent with prior studies^50,74^, we observed pronounced autophagy disruption and protein aggregation. This suggests that even minimal infection is sufficient to alter the local trophoblast microenvironment and trigger stress responses in neighboring healthy cells, a phenomenon also described for other viruses such as human cytomegalovirus^75^.

Furthermore, given the key role of autophagy in maintaining major placental functions, such as CTB differentiation into STBs and facilitating invasion of EVTs into maternal compartment^29,76^, our results show that ORF3a-induced autophagy disruption impairs these critical processes. These may have lasting consequences for pregnancy and fetal outcomes. ORF3a expression reduced hCG-β levels, indicating impaired STB maturation and diminished EVT invasive capacity. Prior work in trophoblast stem cell models has similarly demonstrated that SARS-CoV-2 infection reduced hCG-β secretion and impaired STB maturation^77^. Our findings that ORF3a alone is sufficient to disrupt these processes provide fresh mechanistic insight into how viral proteins may drive placental dysfunction. Such impairment of trophoblast maturation and invasion could plausibly contribute to adverse outcomes observed in SARS-CoV-2–exposed pregnancies, including PE and intrauterine growth restriction.

SARS-CoV-2 is known to compromise epithelial and endothelial integrity by disrupting TJPs in airway epithelial cells and endothelial cells^78^. Autophagy also plays a key role in maintaining epithelial barrier integrity by regulating the recycling and trafficking of TJPs^79^. Our results with Delta virus infection of JEG-3 cells showed mis-localization of the TJP, ZO-1, consistent with barrier disruption. This was further supported by *in vivo* observations in SARS-CoV-2–exposed placentas, where ZO-1 colocalized with ORF3a, underscoring the physiological relevance of this mechanism. Since ORF3a has PBM (SVPL) with known affinity for PDZ-containing TJPs, such as ZO-1, our experimental data with ORF3aWT and ORF3a-NoPBM mutant support the physical association in placental cells where ORF3a-WT is specifically associated with ZO-1and mislocalizes its expression. In addition, pharmacological inhibition of autophagy alone (via bafilomycin) also induced ZO-1 mislocalization, suggesting that ORF3a impact on barrier integrity likely reflects a synergistic effect of both direct ZO-1 interaction and autophagy blockade. This could contribute to the histopathological alterations associated with barrier disintegration observed in placentas from SARS-COV-2 infected pregnancies, including increased syncytial knots and endothelial damage^69,80–82^. Moreover, since ZO-1 plays a role in CTB-STB differentiation^83^, we can speculate that perhaps ORF3a-mediated disruption of ZO-1 may also underlie the reduced differentiation we observed in JEG-3 cells. Impaired barrier integrity and syncytial differentiation are key features linked to adverse pregnancy outcomes such as PE, further suggesting that ORF3a-driven disruption of ZO-1 may contribute to the pathogenesis of these complications in SARS-CoV-2–exposed pregnancies.

In addition to driving protein aggregation, ORF3a-mediated blockade of canonical autophagy shifts the pathway toward secretory autophagy, resulting in increased expression of CD63^+^ EVs. Given the central role of degradative autophagy in clearance of aggregates and maintaining proteostasis, defects in the endolysosomal system can divert autophagy towards a secretory mode^38,39,84^. Our findings suggest that ORF3a exploits this process to activate secretory autophagy and promote EV secretion. Delta variant infection of JEG-3 cells significantly increased CD63 + vesicle production—not only in infected cells but also in neighboring bystander cells. ORF3a expression alone was sufficient to recapitulate this effect: it enhanced CD63 expression, colocalized with CD63 + vesicles, and was itself secreted. These results align with the known role of ORF3a in impairing the endolysosomal system by deacidifying lysosomes, promoting lysosomal and autophagosomal exocytosis, and facilitating unconventional SARS-CoV-2 egress^67,68^.. This is particularly relevant given that viral RNA and proteins, including ORF3a, can persist in the placenta even when systemic maternal infection has resolved. Such EV-mediated propagation of stress and signaling has been implicated in other viral infections^75^ and may contribute to the broader placental dysfunction and inflammatory responses observed in SARS-CoV-2–exposed pregnancies. Moreover, SARS-CoV-2 infection has been shown to enhance exosome secretion^85–87^. Together, these findings support the possibility that even low-level or transient placental infection during pregnancy may leave behind lasting “scars”^88,89^ with long-term and widespread impact on placental function, pregnancy outcomes, and fetal development.

These results are further supported by physiologically relevant hTSC-derived 3D SC-TO models, which provide an important bridge between mechanistic insights from cultured cell lines and associative observations in human placentas. In SC-TOs, constitutive ORF3a expression significantly impaired organoid maturation, consistent with previous studies showing that SARS-CoV-2 infection of hTSCs disrupts STB differentiation^90^. Furthermore, key ORF3a-mediated phenotypes observed in 2D trophoblast cell lines, including autophagy flux blockade and protein aggregation, were robustly recapitulated in ORF3a–expressing SC-TOs. Notably, ORF3a-WT and ORF3a-NoPBM organoids also reproduced the secretory vesicle phenotype, which to our knowledge has not been previously reported, underscoring a direct role for ORF3a in driving this secretory response. Interestingly, the ORF3a-NoPBM mutant showed significant reduction of higher glycosylated forms of CD63 compared to ORF3a-WT, indicating a mechanism through which the PBM motif of ORF3a might affect CD63 association with vesicles. CD63 also has a PBM, which interacts with syntenin-1, promoting exosome formation and trafficking^91^, raising the possibility that ORF3a PBM may functionally intersect with this pathway. Confocal imaging of ORF3a-WT and ORF3a-NoPBM in relation to CD63 further highlights the importance of the PBM motif in mediating ORF3a’s association with CD63^+^ vesicles and its subsequent secretion. These findings position the 3D SC-TO model as a powerful experimental platform to further dissect how viral proteins such as ORF3a disrupt trophoblast function.

In sum, our work provides new insights into potential viral mechanisms through which SARS-CoV-2 infection exacerbates placental dysfunction and could contribute to the clinical complications observed in pregnant women with COVID-19, such as PE. These findings provide a foundation for exploring therapeutic approaches targeting ORF3a, specifically focusing on its PBM region to block its interactions with host cellular proteins and mitigate SARS-CoV-2–associated placental injury

### Limitations of the study

While our findings are supported across multiple placenta models including cell lines, term human placental tissues and 3D organoids, this study has some limitations. The first limitation is using only the Delta variant and its ORF3a protein; since Delta is highly pathogenic, further studies are needed to characterize whether other emerging variants (e.g., NB.1.8.1) and their ORF3a proteins exhibit similar placental effects. Second, while we observed a shift toward secretory autophagy and increased expression of CD63, an EV pathway associated protein, we did not isolate EVs of specific sizes and characterize their molecular cargo or their effects on recipient maternal or fetal cells. Notably, a recent study has demonstrated that SARS-CoV-2- induced EVs isolated from patients with active infection are capable of altering trophoblast differentiation and invasion^87,92^, highlighting the need for deeper mechanistic insights. Our work provides a mechanistic framework for understanding how SARS-CoV-2 may impact placental health and highlights key avenues for future investigation to mitigate the long-term consequences of viral exposure during pregnancy.

## Methods

Human samples were obtained via random sampling from a pre-existing prospective cohort study of pregnant individuals during the COVID-19 pandemic enrolled at Barnes-Jewish Hospital in St. Louis, MO from December 2021-July 2022 (Safety, Testing/Transmission, and Outcomes in Pregnancy with COVID-19 (STOP-COVID-19) study). This study was approved by the institutional review board at Washington University in St. Louis (#202012075). Patients were serially evaluated for exposure to SARS-CoV-2 infection at enrollment. Antepartum infection was evaluated by universal testing at delivery for SARS-CoV-2 using polymerase chain reaction (PCR) and/or antigen testing. The participants provided informed consent to sampling, storage and use of clinical samples. Placenta samples included villous tissue from COVID (+) and COVID (−) patients. The inclusion criteria included term births, singletons, and unvaccinated against SARS-CoV-2. Exclusion criteria included pre-eclampsia, preterm or still births, and any other infections.

### Cell Culture

Human trophoblast cell lines JEG-3 (ATCC HTB-36) and HTR-8/SVneo (ATCC CRL-3271) were propagated in Dulbecco’s Modified Eagle Medium/Nutrient Mixture F-12 (DMEM/F-12, GIBCO, #11330032) enriched with 10% fetal bovine serum (FBS, Gibco, #16140071). The cultures were incubated in a controlled environment at 37°C and an atmosphere containing 5% CO2. JEG-3 cells were induced to undergo syncytialization by treatment with 50 μM Forskolin (Sigma, #F6886) for a duration of 24 hours. For blocking and inducing autophagy pathways in cell lines, bafilomycin A1(Sigma, #B1793) (100 nM) and metformin (Sigma, #317240) (50 μM) were used respectively. hTSC cells (CT30 line) were procured from Thorold W. Theunissen, Washington University in St. Louis. The culture protocols were followed according to previously described detailed methods except a change in substrate coating on six well plates by using Biolaminin 521 (LN521)^62^.

### Plasmids and transfection

SARS-CoV-2 expression plasmids were acquired from Addgene, courtesy of the contribution from Dr. Nevan J. Krogan at the University of California, San Francisco^33^. These constructs are based on the pLVX-EF1alpha vector, fused with a 2xStrep-tag (#141395). The ORF3a-mCherry plasmid, featuring a CMV promoter within a pmCherryN1 vector backbone, was also obtained from Addgene (#165138). The autophagy flux sensor plasmid pRRL-SV40-Puro-CMV-mCheery-EGFP-LC3B was purchased from Addgene (#223712)88. We customized the pLVX-EF1alpha-ORF3a-2xStrep (#141383) plasmid for generating ORF3a-WT and ORF3a-NoPBM mutant. We employed restriction enzyme-based cloning to eliminate the 2x-Strep tag. ORF3a-WT was created by PCR amplifying pLVX-EF1alpha-ORF3a-2xStrep using forward primer with EcoRI (Thermo, ER0271) site and reverse primer with adding stop codon at upstream of the 2xStrep tag sequence followed by BamHI (Thermo, ER0055) site. ORF3a-NoPBM mutant was created using similar forward primer but changing reverse primer by adding a stop codon upstream of the PBM site (SVPL) followed by BamHI site. Post-PCR, the amplicons were subjected to double digestion with EcoRI and BamHI, followed by ligation into the double digested pLVX-EF1alpha vector using T4 TNA ligase (NEB, #M0202S). Ligation mix was transformed into NEB Stable Competent E. coli. Transformed colonies were picked from Luria Bertani (LB) agar plates infused with ampicillin (100mg/ml) (Ampicillin, Sigma, #A5354) and inoculated in overnight cultures of LB media which subsequently harvested for plasmid preparation using Qiagen miniprep kit (Qiagen, #27104). Successful cloning was validated through Sanger sequencing. Trophoblast cells were transfected with plasmids encoding SARS-CoV-2 viral proteins using Lipofectamine 3000 (Thermo, #L3000015) in accordance with the manufacturer’s instructions. All experiments were performed under biosafety level 2 (BSL2) conditions.

### Lentivirus preparation

In 10 cm dishes, HEK293T cells were seeded at a density that would result in 70–80% confluence the next day. TransLentiX (TransLentiX Inc.) was used to perform transfection in accordance with the manufacturer’s instructions. A DNA combination was generated in a 4:3:1 ratio, containing 10 μg of the lentiviral transfer plasmid (ORF3a-WT, ORF3a-NoPBM and pRRL-SV40-Puro-CMV-mCheery-EGFP-LC3B),7.5 μg of the packaging plasmid psPAX2, and 2.5 μg of the envelope plasmid pMD2.G. After adding this mixture and TransLentiX reagent to the cells, they were cultured for six to eight hours. Following incubation, fresh DMEM containing 10% fetal bovine serum (FBS) was added to the transfected medium. After that, the cells were left to generate the virus for 48–72 hours. The lentiviral particle-containing supernatant was then collected, and any cellular debris was removed by filtering it through a 0.45 μm filter. The virus was concentrated using lentiviral concentration reagent from Takara (Lenti-X) as described by manufacturer protocol. Before being used again, the virus particles were separated and kept at −80°C.

### Stable cell line generation

CT30 hTSCs were seeded onto 6-well plates pre-coated with Laminin-521 (BioLamina) and cultured until approximately 70% confluent. Lentiviral particles encoding either SARS-CoV-2 ORF3a-WT or ORF3a-NoPBM mutant were added at 2 μL per well in the presence of 8 μg/mL polybrene (Tocris, #7711). Cells were incubated for 24 hours before replacing the medium with fresh culture medium. After 48 hours, puromycin (Thermo, #A1113803) selection was initiated at a concentration of 2.1 μg/mL and maintained for 5–7 days until all non-transduced cells were eliminated. Stable integration and expression were confirmed by immunofluorescence and Western blot prior to downstream assays.

JEG-3 choriocarcinoma cells were used to establish a stable autophagy flux reporter line. Cells were seeded in 6-well plates and transduced with lentiviral particles encoding the tandem LC3-EGFP-mCherry construct in the presence of 8 μg/mL polybrene. Viral supernatant (2 μL per well) was added and incubated for 24 hours, followed by medium replacement. Puromycin selection (2.1 μg/mL) was applied 48 hours post-infection and continued for 5–7 days. Clonal populations were expanded and screened for robust dual EGFP and mCherry expression using fluorescence microscopy. Functional validation of autophagy flux was performed using known autophagy modulators (e.g., Bafilomycin A1 and Metformin) prior to experimental use.

### 3D SC-TO generation

3D SC-TOs were created using the CT30 hTSC 2D cell line, as previously described^62^. hTSCs were grown in 6-well plates coated with Laminin521. The medium was DMEM/F12 with 0.1 mM 2-mercaptoethanol, 0.2% FBS, 0.5% Penicillin-Streptomycin, 0.3% BSA, 1% ITS-X (Gibco, 51500), 1.5 μg/ml L-ascorbic acid (Wako, 013–12061), 50 ng/ml EGF (Peprotech, AF-100–15), 2 μM CHIR99021 (R&D #4423), 0.5 μM A83–01 (Peprotech, 90943360), and 1 μM SB431542 (BioVision). When the cells reached 80% confluency, they were used to seed 3D SC-TOs according to published protocols^62^. Following dissociation of individual cells using TrypLE, hTSCs were washed twice with Advanced DMEM/F12 and 3000 cells were seeded in a final concentration of 72% Matrigel in Advanced DMEM/F12 before being planted in 30 μL droplets in a 24-well plate. Droplets were incubated for two minutes at room temperature before polymerization for 30 minutes at 37°C. After polymerization, 500 μL of trophoblast organoid media (TOM) was added^62^. Medium was replaced every other day.

### SARS-CoV-2 infection

The SARS-CoV-2 Delta strain (B.1.617.2) was propagated in Vero cells (ATCC CCL-81) under Biosafety Level 3 (BSL-3) conditions at the Baylor College of Medicine. Viral stocks were titrated using a standard plaque assay to determine plaque-forming units (PFU/mL), following all institutional and federal BSL-3 safety protocols and guidelines. For infection experiments, JEG-3 cells and a stably transduced JEG-3-LC3-EGFP-mCherry reporter cell line were seeded in six-well plates. For immunofluorescence studies, cells were plated on glass-bottom plates. Cells were infected with SARS-CoV-2 Delta at a multiplicity of infection (MOI) of 1, using DMEM supplemented with 2% fetal bovine serum (FBS) as the infection medium. After a 2-hour adsorption period, the viral inoculum was removed, cells were washed twice with PBS, and fresh DMEM containing 10% FBS was added. Infected cultures were incubated for 48 hours.

After 48 hours, culture supernatants were collected for viral titration by plaque assay. The remaining cells were either fixed with 4% paraformaldehyde (PFA) for 30 minutes at room temperature for immunostaining or lysed in RIPA buffer for protein extraction. All samples were transferred out of the BSL-3 facility only after confirming complete viral inactivation in accordance with approved safety protocols.

### Invasion assay

For the cell invasion assay, HTR-8 cells were employed to investigate the invasive capacity following transfection with SARS-CoV-2 protein-encoding plasmids. Prior to the assay, cells were cultured under standard conditions in six well plates and transfected for 24 hours with the plasmids of interest. Post-incubation, cells were detached using trypsin-EDTA solution (Gibco, 25300054). The cells were then carefully counted to obtain accurate cell numbers for seeding. Each trans-well insert with precoated Matrigel (Corning, 354480) was placed in 24 well plates according to the manufacturer’s protocol. The number of cells seeded into each insert was standardized to ensure uniformity across experiments. After seeding, the cells were allowed to invade through the Matrigel coating towards a chemotactic gradient provided by the medium containing 20% fetal bovine serum (FBS: Gibco, 16140071) in the lower chamber. The invasion process was conducted under optimal cell culture conditions (37°C, 5% CO2) for a duration of 24 hours to ensure significant invasion while preventing over confluency. At the end of the invasion period, non-invading cells were removed from the upper surface of the membranes using cotton swabs. Cells that had invaded through the Matrigel and reached the lower surface of the membrane were fixed, stained, and quantified. The fixed cells were usually stained with crystal violet (Sigma, C0775), which allows for easy visualization and counting under a microscope.

### Immunofluorescence

Immunofluorescence staining was conducted on human placental tissue, cell cultures, and 3D SC-TOs. Paraffin-embedded fixed placental villous tissue blocks were cut into 5-micrometer-thin sections which further underwent deparaffinization, rehydration, and antigen retrieval in a citrate buffer. The tissue sections were incubated with 1% BSA for blocking and subsequently treated with primary antibodies diluted in 1% BSA targeted to specific antigens for overnight at 4°C, followed by appropriately conjugated secondary antibodies diluted in 1% BSA for 1 hour at room temperature. Nuclei were counterstained with Hoechst-33342 (Invitrogen, H3570) according to the manufacturer’s instructions. After the incubation, sections were washed with PBS and processed for treatment with autofluorescence quenching kit (Vector TrueVIEW, #SP-8400) according to manufacturer instructions. Finally, slides were mounted using VECTASHIELD Vibrance Antifade mounting media provided in the quenching kit and visualized using a ECLIPSE Ti2 (Nikon) confocal microscope.

JEG-3 cells in six well glass bottom plates (Cellvis, P06–1.5H-N) post-transfection were fixed with 4% paraformaldehyde at room temperature for 15 minutes, permeabilized with 0.2% Triton X-100 for 10 minutes and blocked with 1% Bovine serum albumin (BSA) for one hour at room temperature to prevent non-specific binding. The following primary antibodies were used: SQSTM1/p62 (Abcam, ab91526; 1:500), ZO1 (Proteintech, 21773–1-AP; 1:800), Anti-strep tag (Sigma, SAB2702216; 1:300), ORF3a (Cell Signaling Technology, 34340; 1:200), ORF3a (R&D Systems, MAB10706; 1:200) CD63 (Proteintech, 25682-1-AP; 1:300). Secondary antibodies used in the study were Alexa Fluor 488 goat anti-mouse IgG1 and goat anti-rabbit IgG (Thermo, A-11001 & A-11008), Alexa Fluor 594 goat anti-mouse and goat-anti-rabbit (Thermo, A-11005 & A-11012) and Alexa Fluor 647 goat anti-mouse and goat-anti-rabbit (Thermo, #A21237 & #A-21245).

Organoids were immunostained as previously described^62^. Briefly, Matrigel domes were dissociated using Cell Recovery Solution (Corning, #354253) and incubated on ice for 30 minutes. Organoids were washed, fixed in 4% paraformaldehyde on ice, and subsequently washed with PBS + 0.1% BSA. Permeabilization and blocking were performed overnight at 4°C in PBS containing 4% BSA, 5% FBS, and 0.5% Triton-X. Primary antibodies were applied in PBS + 4% BSA + 5% FBS + 0.1% Tween-20 overnight at 4°C, followed by secondary antibodies overnight and later Hoechst staining. Organoids were mounted using Prolong Gold Antifade (ThermoFisher #P36930) and imaged on a Nikon ECLIPSE Ti2 confocal microscope.

### Proteostat staining

Cultured JEG-3 cells were fixed using 4% paraformaldehyde for 15 minutes at room temperature for proteostat aggresome detection. Simultaneously, paraffin-embedded sections, rehydrated and deparaffinized, were prepared alongside. Both fixed cells and tissue sections were permeabilized with0.2% Triton X-100 and blocked with 1%BSA to prevent non-specific staining. The Proteostat aggresome (Enzo life sciences Inc, ENZ-51035–0025) staining proceeded as per the manufacturer’s guidelines, with an emphasis on protecting the samples from light. Proteostat dye was added at a 1:2000 dilution for 30 min at room temperature. Post-staining, cells and sections were washed, counterstained with Hoechst-33342 for nuclear visualization, and mounted using Prolong gold anti-fade mounting media for fluorescence microscopy.

### Western blot

Post-transfection, JEG-3 cells were lysed after a 24-hour period using RIPA lysis buffer (Thermo, 89901), supplemented with cocktails of protease and phosphatase inhibitors for protein preservation. Protein concentrations were determined via the Bicinchoninic Acid (BCA) method (Thermo, 23225).

Protein extraction from organoids was performed using snap freezing in liquid nitrogen method. Organoids were recovered from Matrigel dome by trituration in Cell Recovery Solution (Corning, #354253) and incubated in ice for 30 minutes. Subsequently washed with PBS 2 times and organoid pellet collected which immediately snap-frozen in liquid nitrogen. The frozen pellet was mechanically broken using pipette tip and resuspended in 20 mM TRIS buffer (pH −8.0) containing protease inhibitor cocktail (Thermo, A32963). The suspension further dissociated using sonication (3 cycle on and 10 cycle off) for 3 minutes. Subsequently centrifuged and supernatant collected and proceeded for BCA protein concentration estimation.

The lysates were then subjected to electrophoresis on 4%−20% gradient Mini-PROTEAN TGX precast gels (Bio-Rad Laboratories, 4561093) for resolution of the proteins. Subsequently, proteins were electroblotted onto Polyvinylidene Difluoride (PVDF) membranes (Sigma, 05317–10EA) at a constant 60 volt for two hours. Following the transfer, membranes were blocked to prevent non-specific binding and incubated with the relevant primary antibodies at 4°C overnight. The following day, membranes were thoroughly rinsed three times with 0.1% Tween-20 in Tris-buffered saline (TBST) before a 1-hour room temperature incubation with IR dye-linked secondary antibodies. After a final washing step to remove unbound antibodies, protein bands were visualized on a ChemiDoc imaging system (Bio-Rad Laboratories), using infrared detection wavelengths. Band intensities were measured with Image Lab software (Bio-Rad), and data were normalized against housekeeping proteins to ensure accuracy in protein quantification. The following antibodies were used in Western blot: GAPDH (Cell Signaling Technology, 97166S & 5174S; 1:3000), LC3 (Novus, NB600–1384; 1:1000), SQSTM1/p62 (Abcam, ab91526; 1:2000), human Chorionic Gonadotropin (Abcam, ab9582; 1:500), CD63 (Proteintech, 25682–1-AP; 1:1000).

### Statistical Analyses

All statistical analyses were conducted using GraphPad Prism 9. The Shapiro-Wilk test was employed to verify the normal distribution of continuous variables. Pairwise comparisons were evaluated for statistical significance using either the Student’s t-test or the Mann-Whitney test, depending on normality. For comparisons involving three or more groups, a one-way ANOVA was utilized, followed by Tukey’s test for multiple comparisons. In cases of nonparametric data distribution, the Kruskal–Wallis test was applied, along with Dunn’s test for post-hoc analysis. Detailed descriptions of the statistics, tests used, and post-hoc tests for multiple comparisons are included in the legends of each figure and their source data.

## Supplementary Material

Supplementary Files

This is a list of supplementary files associated with this preprint. Click to download.

- SupplementaryMaterialFinal.pdf

## Data Availability

Further information and requests for resources and reagents should be directed to and will be fulfilled by the lead contact, Indira Mysorekar (Indira.Mysorekar@bcm.edu)
